# Recycled Materials in Civil and Environmental Engineering

**DOI:** 10.3390/ma15113955

**Published:** 2022-06-02

**Authors:** Andrea Petrella, Michele Notarnicola

**Affiliations:** Department of Civil, Environmental, Land, Building Engineering and Chemistry, Polytechnic University of Bari, Via E. Orabona, 4, 70125 Bari, Italy; michele.notarnicola@poliba.it

## 1. Introduction

Waste represents a huge reserve of resources that, after appropriate management, can guarantee a sustainable and continuous supply of materials and energy over the years [1,2,3,4].

Waste management includes the activities aimed at managing the entire waste process which involves the collection, transport, treatment (recovery or disposal), reuse, and recycling of waste materials in order to reduce the effects on human health and the impact on the environment [5,6,7].

The proper management of hazardous and non-hazardous waste deriving from civil and industrial operations is the basis of the principles of Circular Economy that the European Union has indicated in specific Regulations and Directives devoted to the control of the entire waste cycle, from production to disposal, with specific attention to recovery and recycling.

In this respect, the aim of this collection of papers was to report recent innovative studies based on the use of secondary raw materials for applications in Civil and Environmental Engineering. 

For the purpose, eleven papers were related to the preparation of innovative construction materials, whereas six papers were related to the treatment of wastes for environmental applications.

The investigations were characterized by a common purpose, i.e., to find a way to reduce the amount of waste generated, thus reducing the need for landfilling and optimising the values of these novel materials which become an abundant resource that can be easily reused for different applications.

## 2. Recycled Waste for Construction Materials

In the papers by Petrella and coworkers [8,9], industrial and agricultural by-products such as end-of-life tire rubber and wheat straw were used as aggregates for the production of unconventional cement mortars prepared by a cheap and environmentally safe process. The artifacts resulted in thermal insulating with respect to the sand-based references, while the presence of another aggregate as perlite was used to improve the mechanical strengths with no detrimental effects on the thermal conductivities due to the porous nature of the inorganic material [10,11]. The conglomerates with end-of-life tire rubber also showed hydrophobic behavior due to the low water absorption, while the conglomerates with wheat straw showed high impact resistance and acoustic absorption. Non-structural insulating products, specifically for indoor applications, may be a possible use of these composites.

Tsai and coworkers [12] presented a trend analysis on the generation and treatment of industrial waste in the 2010–2020 period, with specific reference on promotion policies and regulatory measures for mandatory renewable resources from industrial sources in Taiwan. To the purpose, the renewable resources considered in the present study were reclaimed asphalt pavement (RAP) material, water-quenched blast furnace slag, and ilmenite chlorination furnace slag for the production of recycled aggregate materials in concrete and construction applications.

The paper by Stevulova and coworkers [13] was focused to study the properties of sustainable cement mortars with cellulose fibers derived from waste paper recycling operations. This represents an economic, technically feasible, and ecological approach to waste management for indoor applications. Moreover, the study showed the benefits of these innovative composites for providing healthy living solutions, due to the cellulose aggregate property to regulate humidity inside buildings.

The paper by Dilbas and coworkers [14] showed the influence of treatment and mixing methods on recycled aggregate concretes (RAC) obtained after various techniques. Specifically, a statistical study was carried-out after evaluation of the compressive strength test results of concretes designed with Absolute Volume Method (AVM) and Equivalent Mortar Volume Method (EMV) and containing natural aggregate (NA), recycled aggregate (RA), and silica fume (SF). 

The paper by Karimipour and coworkers [15] studied steel-concrete-steel (SCS) sandwich panels based on two thin high-strength steel plates separated by a core made of low-density and low-strength thick concrete. The panels were characterized by a new stud-bolt connector able to regulate its shear behaviour, and the bolts’ diameter, concrete core’s thickness, and bolts’ spacing were analysed. Furthermore, the core was realised with normal-strength concrete and steel fibre concrete (SFC) and with recycled coarse aggregate. It was observed that increasing the stud-bolts diameter or using SFC improved the shear strength and ductility ratio of the sandwich panels.

The paper by Pederneiras and coworkers [16] studied the physical and mechanical properties of environmentally friendly and low-cost products based on coir fibers in rendering mortars with two different binders. It was observed that the addition of coir fibers led to improvements in the cracking behavior and that the volume fraction of the fibers and the binder used were the main factors which improved the brittleness of the conglomerate. A cement-lime binder together with higher fiber content determined the highest fracture toughness, thus showing the potential feasibility of these fibers for reinforcement of cement and cement-air-lime mortars.

In the work by Poulose et al. [17], HDPE/strontium aluminate-based auto glowing composites (SrAl_2_O_4_: Eu, Dy (AG1) and Sr_4_Al_14_O_25_: Eu, Dy (AG2)) were prepared and the phosphorescence, thermal, mechanical and rheological characteristics were analysed. Due to the better mechanical characteristics and long afterglow time of these HDPE composites with the addition of the AG1 and AG2 fillers, applications in roadway nighttime displays, fluorescent lamps, etc. can be considered.

In the paper by Rocha et al. [18], waste tire steel fibers (WTSF) were used for the production of soil-cement blocks. Specifically, threefold mixtures with 10% by weight of Portland cement and with 0%, 0.75%, and 1.5% volumetric additions of WTSF were prepared and characterized. The soil-cement blocks with 1.5% WTSF showed an average compressive strength increase of 20%. It was observed that all the results of the investigated samples were in agreement with the minimum requirements of the different standards considered. 

The paper by Deb et al. [19] was devoted to the use of recycled brick aggregates in the preparation of pervious concrete (PC) which were characterized by mechanical tests together with an analysis of pore structure distribution. Replacement ratio and particle size were investigated on structural performance and pore feature through laboratory testing and image processing techniques.

In the paper by Khan et al. [20], the compressive strength of recycled aggregate concrete was predicted by the use of machine learning techniques like decision tree and two approaches as gradient boosting and bagging regressor. Correlation coefficients and statistical tests were carried out to evaluate the performance of each method and the validity was confirmed by k-fold evaluation and error dispersals.

## 3. Recycled Materials for Environmental Applications

In the paper by Valentukeviciene and coworker [21], sorbents from agricultural and cement manufacturing waste were used to remove oil residues in wastewater with a turbidity removal efficiency of 64%, and color removal efficiency of 56%, and total iron concentration removal ~68%. Moreover, straw sorbent oil adsorption capacity was up to 33 mg/g, peat sorbent 37 mg/g, and mineral sorbent 1.83 mg/g, demonstrating the effectiveness of these recycled sorbents as environmentally sustainable materials for wastewater treatments.

In the review by Borjan and coworkers [22] different existing recycling technologies for carbon fiber-reinforced composites were presented with advantages and drawbacks. This is because the production of these materials has dramatically increased in the last years together with the quantity of waste. Specifically, the study was focused on chemical recycling using sub- and supercritical fluids which were demonstrated to be more efficient systems to recover clean fibers characterized by good mechanical properties. 

In the paper by Medynska-Juraszek and coworkers [23], wheat straw, sunflower husk, and pine-chip biochars produced by torrefaction/pyrolysis conditions were chemically modified with ethanol and HCl for applications as Na^+^ sorbents from aqueous solution. Wheat straw biochar, obtained by pyrolysis (550 °C), showed the best Na^+^ sorption capacity. The pre-treatments with EtOH and HCl showed an improvement in sorption capacity in biochars produced through torrefaction (<300 °C). Moreover, the biochars produced by torrefaction may be used for Na+ removal with lower energy demand and carbon footprint by ethanol treatment, resulting in a very promising method for applications in Environmental Engineering.

In the review by Amari and coworkers [24] different types of clay-polymers nanocomposites (CPNs) were studied together with their efficiency in the removal of different pollutants from water/wastewater by flocculation and adsorption. Specifically, the ability of these novel and cost-effective adsorbents to remove bacteria, metals, phenol, tannic acid, pesticides, dyes, etc., was reported. The adsorption capacity was higher than bare clay and polymer adsorbents together with a better regeneration. 

In the paper by Irtiseva et al. [25], three granular sorbents from secondary raw materials were produced and studied for the sorption of diesel fuel and motor oil. Specifically, homogenised peat, waste tyre rubber, and fly ash cenospheres were used. The granules’ morphology, porosity, mechanical properties, and sorption kinetics were analyzed. Moreover, the hydrophobicity and the sorption capacity of the pellets were determined according to the type of oil product.

In the review by Loffredo [26] new technologies of biomass conversion were developed with the primary goal of recycling biowaste and producing bioenergy. The co-products and by-products of these processes such as biochar, hydrochar, and digestate showed physicochemical properties that suggested their use as biosorbents of phenolic and steroidal environmental estrogens in wastewater treatment and soil remediation. The review presented recent advances in the characterization of these materials and qualitative and quantitative aspects of the adsorption/desorption of these pollutants, including data modeling.
